# Use of Minimal Amounts of Hyaluronidase in the Ultrasound-Guided Treatment of Acute Vascular Occlusion by Hyaluronic Acid: A Preliminary Report

**DOI:** 10.1093/asjof/ojae025

**Published:** 2024-04-23

**Authors:** Urso Simone Ugo, Molinari Paola, Fundarò Salvatore, Mosti Giovanni

## Abstract

**Background:**

Complications from hyaluronic acid (HA) fillers are increasingly frequent, given the very high number of treatments performed every day worldwide. They are routinely treated with high doses of hyaluronidase, which can cause significant local and general unwanted effects.

**Objectives:**

The aim of our work is to demonstrate that when the origin of the complication is precisely identified and treated under ultrasound guidance, a few hyaluronidase units are enough to treat it effectively.

**Methods:**

Five young female patients came to our observation for vascular lesions from injection of HA fillers performed in the immediately preceding days, in 4 cases, and a few weeks earlier in the fifth case. All lesions were accurately identified by ultrasound and treated with hyaluronidase.

**Results:**

The 4 promptly treated patients fully recovered with a hyaluronidase dose of 87 ± 44 IU (range, 30-150 IU). The fifth patient, treated later, markedly improved regarding clinical picture and symptoms. No early or late side effects have been reported from this hyaluronidase dosage.

**Conclusions:**

Our data confirm that if the lesion at the origin of the skin damage is precisely localized with ultrasound examination and treated under ultrasound guidance, a few units of hyaluronidase, injected directly into the HA accumulation, effectively resolve the skin damage. At the doses we used, which were much lower than those usually recommended, hyaluronidase proved to be not only effective but also free of any side effects.

**Level of Evidence: 5:**

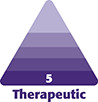

The administration of hyaluronic acid (HA) fillers in aesthetic medicine is the second most common nonsurgical procedure.^1^ HA is a high-molecular-weight polysaccharide that is easily absorbed. Because of its physiological pH and rheological characteristics, it is considered an extremely safe treatment.^2,3^ Despite the generally reported safety, we may find several papers in the medical literature highlighting complications, sometimes serious, related to the HA injections.^4-9^ The filler injection in high-risk areas or a deep injection at the periosteal level can expose the injecting doctor to the risk of vascular complications, sometimes serious ones, which can lead to tissue necrosis and, rarely, blindness.^10^ However, hyaluronidase, an enzyme that induces HA depolymerization, can dissolve its accumulation. Injecting hyaluronidase into the area previously treated with fillers can significantly reduce the severity of complications, especially if early recognized, and even more when the injection is guided by ultrasound and then targeted to the area of interest. An injection targeted at the accumulation of the filler causing the complication, lets the doctor inject hyaluronidase at doses much lower than those commonly recommended and such as to avoid complications produced by the hyaluronidase itself.^11,12^ The ultrasound examination is very useful to carry out a preliminary assessment of the region to be treated and to avoid HA injections at the points at risk. At the same time, it is also beneficial for ultrasound-guided treatment of vascular complications, allowing low-dose hyaluronidase injections.^13^ This work aims to demonstrate that an ultrasound examination can precisely evidence the vascular damage induced by filler injection and make the resolution of such damage possible with a minimal hyaluronidase amount.

## METHODS

**Figure 1.**
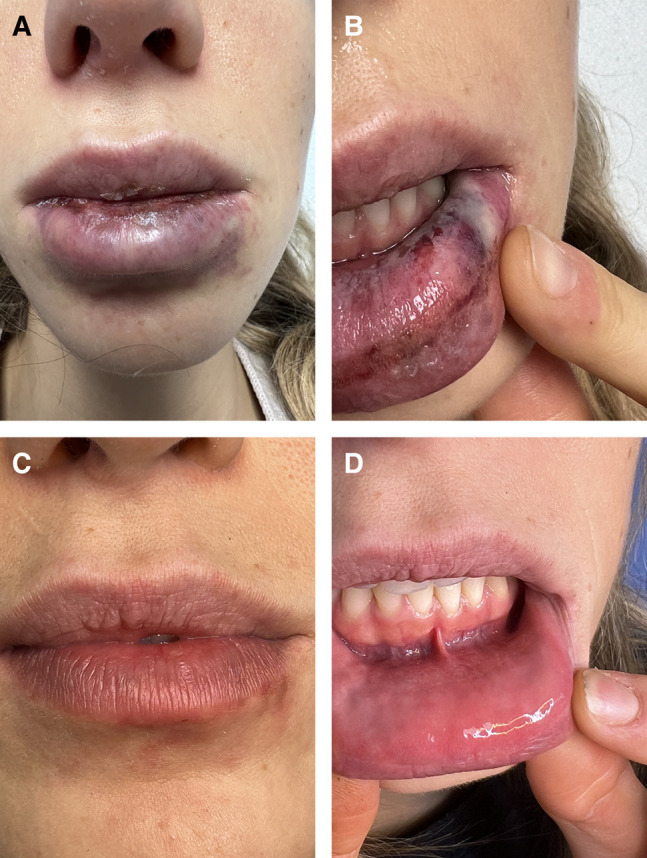
A 26-year-old female, complaining of inferior lip vascular compromise. (A) Three days after hyaluronic acid (HA) injection, lip outer side, (B) 3 days after HA injection, lip inner side, (C) lip outer side, 20 days after hyaluronidase treatment, and (D) inner side, 20 days after hyaluronidase treatment.

**Figure 2.**
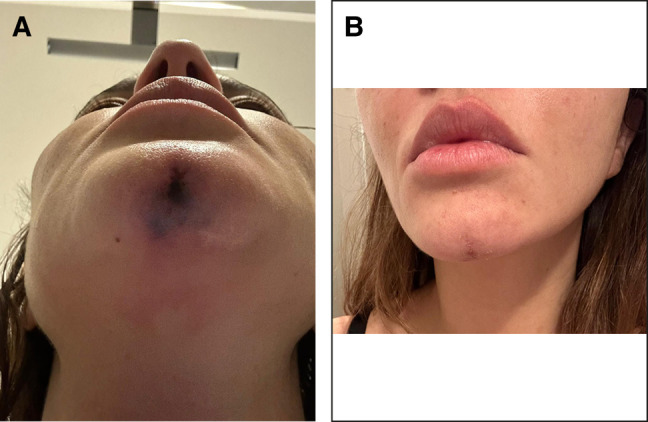
A 34-year-old female, complaining of skin vascular compromise at chin level. (A) One day after hyaluronic acid injection, and (B) 20 days after hyaluronidase treatment.

**Figure 3.**
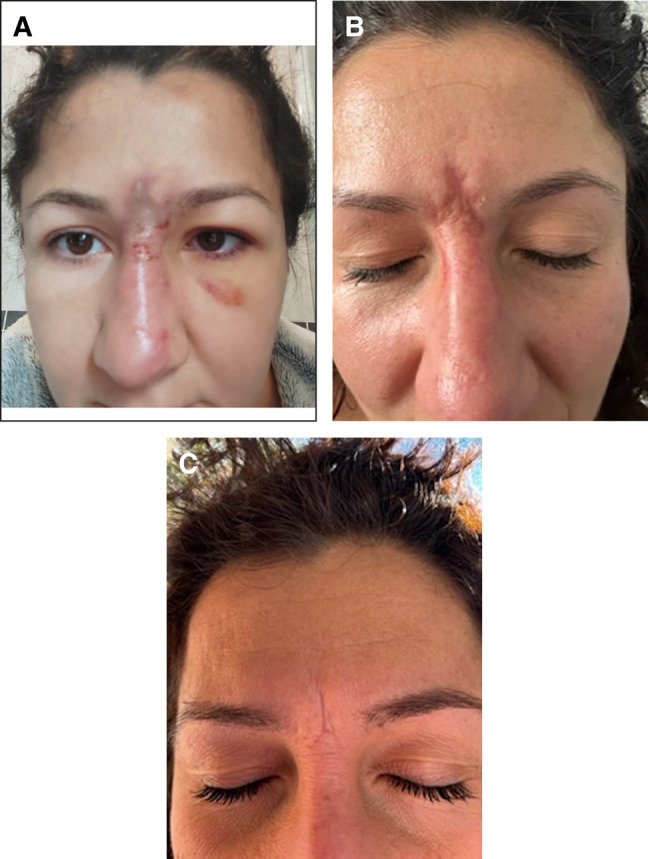
A 38-year-old female, complaining of skin lesion at the nose and glabella. (A) Self-made picture showing the skin damage a few days after hyaluronic acid (HA) injection, (B) when she came for consultation, 38 days after HA injection, and (C) 20 days after hyaluronidase treatment.

From December 2022 to June 2023, 5 female patients aged between 26 and 38 years (mean 30.5 ± 3.2 years) with acute occlusion of face vessels after administration of HA fillers came to our observation (Table 1). Four patients were seen 4.5 days (range, 1-6 days) after treatment, and 1 much later, 38 days after treatment. Regarding the type and location of the lesions, the first patient had vascular compromise of the left lateral lower lip mucosa because of damage to the left inferior labial artery (Figure 1A, B). The second patient had a small area of skin necrosis in the mental region (Figure 2A) because of damage to of the left submental arteries. Patients 3 and 4 showed tissue distress with inflammation and pustules formation. In Patient 3, these lesions occurred on the right side of the nasal bridge because of the nose’s right dorsal artery involvement. In Patient 4, the skin lesion occurred in the left lateral superior prolabium area with partial involvement of the nasolabial groove because of the involvement of branches from the left angular artery. In Patient 5, the damage was the final result of skin necrosis, with a stabilized skin lesion extending from the lower right corner of the glabella to the lateral nasal bridge because of the lesion of the branches of the right dorsal artery of the nose and branches of the right supratrochlear artery (Figure 3A, B).

**Table 1.** Patients' Characteristics

Patient	Gender	Age	Complained damage	Position	Days after HA injection	Hyaluronidase (IU)	After 20 days
1	F	26	Lip necrosis	Inferior lip	3	150	Healed
2	F	34	Skin necrosis	Chin	1	75	Healed
3	F	31	Tissue distress and pustules	Nose-labial	2	100	Healed
4	F	32	Tissue distress and pustules	Nose	2	80	Healed
5	F	38	Skin scar resulting from necrosis	Glabella/Nose	38	30	Stable lesion

Four patients reported pain and a severe state of anxiety. Patient 5 reported, in addition to the severe emotional discomfort caused by the skin lesion, a sense of weight in the glabella/nose area. During the first consultation, all patients underwent a thorough medical history (especially for allergic diathesis to drugs) and an ultrasound diagnosis. All of them were treated by galenic hyaluronidase (ready-to-use and undiluted) injections, ultrasound-guided, by 1 cc syringes with a 30G 25 mm needle. An initial bolus was administered in the area of the most significant HA accumulation associated with arterial flow impairment, as evidenced by Doppler flowmetry. When the treatment was incomplete, further hyaluronidase injections were given until a clear improvement in blood flow was achieved. During the initial treatment period, oral antibiotic therapy was prescribed for 5 days as a single daily dose. The 4 early patients received antiplatelet therapy (acetylsalicylic acid 300 mg once a day orally) for 2 weeks to prevent further clot formation because of vascular damage.^14,15^ All patients underwent a new ultrasound examination the day after treatment and after a further 20 days.

This work is a retrospective report on the treatment of vascular complications because of the wrong injections of HA filler. Hyaluronidase treatment is the only one widely approved treatment for these complications and is considered urgent and mandatory. For this treatment, no ethical approval is requested in Italy. All patients consented to the treatment by signing an informed consent. All treatments were performed in adherence with the Declaration of Helsinki and in accordance with the standards of good clinical care following local guidelines and regulations.

## RESULTS

**Figure 4.**
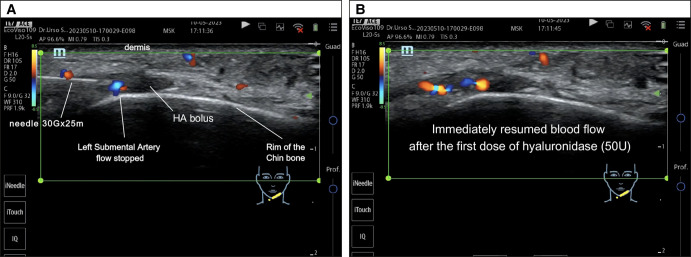
(A) Ultrasound examination showing a hyaluronic acid bolus blocking the left submental artery flow. Injection of hyaluronidase 50 IU, ultrasound-guided, directly into the bolus. (B) Ultrasound examination showing the immediate flow resumption following hyaluronidase injection.

The patients were treated with a mean hyaluronidase dose of 87 ± 44 IU, ranging from 30 to 150 IU. A partial improvement in blood flow in the treated area was observed in the first 4 patients after the first administration of 30 IU (Figure 4A, B, Video). The treatment was completed in the same session, with subsequent administrations between 35 and 50 IU, for a maximum of 150 IU. On the following day, an additional treatment was necessary in 2 cases (Patients 1 and 4) because of persistent skin lesion, although improved. The ultrasound examination still showed a small HA accumulation surrounding the vessel. In both cases, the treatment was completed with a single administration of 30 IU, bringing the total dose of hyaluronidase to 150 IU in 1 patient and 80 IU in the other. In the patient who came late to see us, the initial bolus of 30 IU of hyaluronidase in the area of residual HA accumulation was enough to solve the skin injury at the root of the nose near the emergence of the right supratrochlear artery and to relieve the sense of weight in the forehead.

At the 20-day control, the first 4 patients showed complete tissue healing (Figures 1C, D, 2B). The fifth patient showed a significant improvement in her skin lesion (Figure 3C). The pain and weight feeling on the forehead completely disappeared. Moreover, the ultrasound examination showed an impressive flow improvement in the affected area.

## DISCUSSION

With the widespread use of aesthetic medical treatments based on HA filler injections, there has been a parallel increase in complications, both acute and late, with variable severity grades. The most feared complication, luckily quite rare, is the vascular complication, which occurs through 3 mechanisms:

1. direct intraarterial injection of the filler;
2. vessel compression because of an excess of product injected into a confined space; and
3. arterial spasm.

The mechanism triggering the vascular spasm is unknown. It could be a critical perfusion pressure reduction, a reduced oxygen tension, or a reaction to some substance released into the tissues because of ischemia. The consequences of vascular complications can be devastating, ranging from permanent tissue damage, ischemia-related, to blindness, of which 98 cases have been described. The glabella, the nasal region, the nasolabial folds, and the forehead are the areas most at risk for ocular complications. Arterial occlusion can occur either anterogradely or retrogradely, depending on the pressures involved in the injection. Ultrasound analysis has been established as an essential diagnostic technique for evaluating vascular complications because of the fillers used. Although unable to identify the precise pathophysiological mechanism (intraarterial injection, vascular compression, or spasm), the ultrasound examination has proved to be a considerably precise tool in detecting vascular impairment, providing a detailed assessment of the area under examination, of the filler accumulation (responsible for the complication), and of the blood flow changes. Ultrasound control has also proved extremely useful for performing an ultrasound-guided, targeted, and precise hyaluronidase treatment, making low-dose administration possible. Current guidelines recommend the immediate use of high doses of hyaluronidase without ultrasound guidance to allow the drug to “flood” the treated territory and penetrate the occluded vessel wall. This procedure should be repeated at 1 h intervals, with an administration of 500 IU for each area of about 3 cm². Recommended doses range from 500 IU for treating small skin lesions up to 1500 IU for larger areas,^14,15^ but treatments with 4500 IU hyaluronidase have also been reported.^16^ It should be noted that this approach, although supported by the literature,^17^ is not consequence free. It is mainly based on the visual assessment of the damage and the experience of the injecting doctor. However, the visual identification of the occluded vessel and especially of the origin of the complication is always tricky, if not impossible. Not even the specific area of filler accumulation responsible for the occlusion can be pinpointed precisely. Although the hyaluronidase treatment is generally believed to be safe and free of dangerous side effects, it is necessary to consider that there are different types of hyaluronidases on the market and that not all of them are the same in terms of safety and absence of side effects. In a recent publication, bovine-derived hyaluronidase was shown to be very irritating to tissues, causing redness and swelling, whereas hyaluronidase obtained with recombinant technology was the safest. In addition, doses above 300 IU significantly increased eosinophils in the treated area, predicting the possible onset of allergic reactions.^16^ Indeed, it has been reported that high doses of hyaluronidase can cause significant allergic reactions up to anaphylactic shock (rare) and local inflammatory reactions that are particularly bothersome for patients even in the following weeks. In addition, when injected into the bloodstream to treat periocular lesions, hyaluronidase proved toxic to the retina.^2,18-21^ High or very high dosages are regularly used in the nonultrasound-guided treatment of vascular complications and, sometimes, for the resolution of nodules, granulomas, and other aesthetic lesions because of the fillers.^16,22^ It has been described that the incidence of adverse reactions is dose dependent.^23^ As a consequence, it is crucial to be able to inject the minimum effective dose^24,25^ to avoid or, at least, minimize their occurrence. In addition, it should not be overlooked that hyaluronidase is a drug and, as with all drugs, the minimum effective dose must be recommended to minimize possible side effects.

The ultrasound approach offers not only real-time control of the progress of treatment but also allows accurate monitoring of tissue response and patient evolution throughout therapy.

Administration of low doses of hyaluronidase is possible when it is carried out under ultrasound guidance. In the cases reported in this study, ultrasound-guided injection of a very low dose of hyaluronidase carried out precisely in the area of interest provided immediate positive results in the 4 patients with acute complications. In the fifth case, characterized by a late treatment after HA injection, there was a significant improvement of the skin injury and of the patient-reported subjective symptoms. Our results represent a further confirmation of previously published data.^15^ Therefore, it is difficult to understand why some authors still suggest potentially harmful high doses of hyaluronidase, even for ultrasound-guided injections.^26^ Hyaluronidase did not cause any side effects at the doses we used.

A weak point of our work is the small case series. However, it should be noted that the results obtained with low doses of hyaluronidase were consistent and repeated in all our patients. They further confirm what has already been reported in the literature.

## CONCLUSIONS

Ultrasound is a fast, safe, and reliable method that allows the doctor to promptly detect complications related to the use of fillers, including the risk of vascular events. Ultrasonography is also very useful in treating complications. It makes it possible to inject ultrasound-guided doses of hyaluronidase significantly lower than those recommended by guidelines without ultrasound guidance. Ultrasound-guided administration of a few units of hyaluronidase may prove advantageous in reducing complications related to this enzyme that appear to be dose dependent. Using an ultrasound device is an essential tool in the practice of modern aesthetic medicine.
